# Anxiety and Depression in Patients With Physical Diseases and Associated Factors: A Large-Scale Field Survey in General Hospitals in China

**DOI:** 10.3389/fpsyt.2021.689787

**Published:** 2021-07-28

**Authors:** Wanlin Yang, Ling Xiao, Zhiyong Yuan, Huan Huang, Yilei Xiang, Zhongchun Liu, Cai Nan, Huiling Wang, Gaohua Wang

**Affiliations:** ^1^Department of Psychiatry, Renmin Hospital of Wuhan University, Wuhan, China; ^2^Hubei Institute of Neurology and Psychiatry Research, Wuhan, China; ^3^Computer College of Wuhan University, Wuhan, China; ^4^Hubei Provincial Key Laboratory of Developmentally Originated Disease, Wuhan, China

**Keywords:** anxiety, depression, physical diseases, general hospitals, risk factors

## Abstract

**Introduction:** To investigate the characteristic of anxiety and depression among patients in general hospitals, and explore the degree of the clinical symptoms and correlated social economic factors.

**Methods:** This is a cross-sectional survey of anxiety and depression in patients with physical diseases, who were suspected of depression and anxiety based on their clinical performance by their physicians and PHQ ≧ 8, from various clinical departments of 57 general hospitals in China. Data regarding demographic characteristics and clinical characteristics were collected. Social and psychological factors and the severity of anxiety or depression were collected through self-rating scales. Finally, we used multivariate logistic regression to identify the factors associated with anxiety and depression in patients with physical diseases.

**Results:** A total of 2,105 (84.6%) valid and completed questionnaires were returned. The proportion of anxiety, depression, combined depression and anxiety, either anxiety or depression among the patients with physical diseases from all clinical departments was 63.3, 75.1, 57.1, and 81.2% respectively. Further regression analysis indicated that gender, monthly income, specific physical diseases, personality traits, social supports and life negative events were related factors of both anxiety and depression.

**Conclusions:** Anxiety and depression were common in patients with physical diseases, with a high proportion of co-morbidity of anxiety and depression. Females, patients with cancer, poor social support and negative life events reported more severe anxiety and depression. The results may help to understand the present situation of anxiety and depression in general hospitals in china, and identify the patients with high risk of depression and anxiety.

## Introduction

Anxiety disorders and depressive disorders are two most common mental disorders. Depressive disorder is mainly characterized by significant and lasting low mood, and its core symptoms are low mood, pleasure and energy decline or lack. More than 264 million people suffer from depression worldwide (1). A survey in China showed that the lifetime prevalence of depressive disorder was about 5.8–7.8% (2). In high-income countries, the link between suicide and depression has been well-established (3). The main clinical manifestations of anxiety disorders include anxiety, tension, worry and fear. A recent China Mental Health Survey conducted a cross-sectional epidemiological survey of the prevalence of mental disorders from July 22, 2013 to March 5, 2015 in 31 provinces across China, and results showed that anxiety disorder was the most common mental disorders, with a lifetime prevalence of 6.3–8.8%. Approximately one-third of patients with mental disorders have some form of anxiety disorder, and its financial burden ranked first in all mental disorders (2). Furthermore, the 2019 Global Burden of Disease Survey found that mental disorders had a large contribution to disability (4). And the comorbidity of anxiety and depressive disorders is common. A large cohort study in Netherlands showed that 67% depressive patients had a current comorbid anxiety while 63% anxious patients had a concurrent depression (5).

Although there are effective treatments for mental disorders, A study in 2007 indicated that between 76 and 85% of patients in low- and middle-income countries have not received any care or treatment for their mental disorders (6). Moreover, undertreatment is popular in patients diagnosed with anxiety or depression in China. And in patients who seek for services, 68.5% choose general medicine service, nearly 3.8 times higher than mental health speciality service (18%) (7). That means patients with anxiety or depression tend to visit general hospitals for medical care, furthermore quite a number of the patients believe that they are suffering from physical diseases rather than mental disorders. Moreover, many studies indicated that depression and anxiety are common psychological problems in Chinese inpatients in general hospitals (7). Previous survey showed that among the pulmonary hypertension patients, the prevalence of depression and anxiety were 21 and 24% (8). And a study of COPD patients showed 31 and 13% prevalence of anxiety and depression (9). Shinkov et al. found that depression and anxiety were more prevalent in the subjects with known diabetes than in those with normal glucose tolerance (10). Götze et al. found that cancer survivors were more depressed and anxious than the general population, while moderate to severe depression and anxiety were reported to be 17 and 9%, respectively (11). According to the survey of Williams et al., the prevalence of post-stroke depression was 26%, and post-stroke anxiety was 30% (12). It is shown that patients with physical diseases were often accompanied by or secondary to depression or anxiety disorders (9–11, 13). Our former research suggested that the non-psychiatric clinicians and nurses in Chinese general hospital were short of psychiatry related training, and most of the clinicians could not understand the manifestations of anxiety and depressive disorders, or how to make diagnosis for patients with depressive or anxiety disorders (14). And the untreated anxiety or depressive symptoms in the patients may influence the treatment and prognosis of physical diseases, or even cause serious consequences, such as suicide.

### Aims of the Study

In order to know about the characteristic of anxiety and depression in general hospitals, the present study made preliminary screening of patients with somatic diseases in general hospitals, and for those who were suspected of depression and anxiety based on their clinical performance by their physicians and PHQ ≧ 8, further investigate the degree of their clinical symptoms, correlated social economic factors. Our research was the first multi-center study, including over 50 general hospitals from four provinces in China. Our result was a representative sample of general hospitals in China. The results may help to understand the present situation of anxiety and depression in general hospitals in china, and identify the patients with high risk of depression and anxiety.

## Methods

### Subjects

This cross-sectional study was conducted from April 2013 to December 2014 in 57 general hospitals located in four provinces, including Hubei (central China), Zhejiang (eastern China), Heilongjiang (northern China) and Yunnan (southern China). The survey used a consecutive sample of 2,381 outpatients and inpatients from all the clinical departments of the hospitals. The inclusion criteria include: age from 12 to 80, Chinese Han nationality, educational level above 5 years, ability to understand and cooperate to complete the assessment, score ≧ 8 and suggested possible depression or anxiety in primary screening with Hospital Anxiety and Depression Scale (HADS) (15). The exclusion criteria include: past history of any mental disorders according to ICD-10 Diagnostic Criteria for Mental and Behavioral Disorders, patients in end stage or very frail. There were a total of 2,105 valid responses.

The Ethics Committee of Renmin Hospital of Wuhan University approved the study protocol (No. 2012-031). All participants completed informed written consent prior to their enrollment in the study.

### Data Collection

The demographic data were collected, including age, gender, marital status (married, divorced/widowed, single), level of education (junior high school or below, senior high school or technical secondary school, bachelor degree or junior college, master degree or above), and monthly income. We selected patients whose score ≧ 8 with Hospital Anxiety and Depression Scale (HADS), suggested possible depression or anxiety in primary screening. In addition, we collected detailed information about the physical disease of the participants, including diagnoses (multiple diseases, circulatory, neurological, digestive, endocrine, urogenital, musculoskeletal, respiratory diseases, cancer, and other diseases), duration, and severity (mild, moderate, and severe). Multiple diseases means combined with several chronic diseases, such as hypertension, coronary heart disease and diabetes etc.

### Questionnaire

The Self-Rating Anxiety Scale (SAS) (16) and Self-Rating Depression Scale (SDS) (17) were used to assess subjective feelings of anxiety or depression respectively. These two scales have better reliability and validity, which were widely used in clinical studies. The SAS questionnaire contained 20 items consisting of four grades, with questions based on feelings of anxiety and mood in the previous 7 days. A score of 50 or more on the SAS categorized individuals as suffering from anxiety, 50–59 categorized as mild anxiety, 60–69 categorized as moderate anxiety, and 70 or more categorized as severe anxiety (18). The SDS questionnaire consists of 20 items, of which 10 items indicate negative experience and 10 items indicate positive experience. A score of 53 or more on the SDS categorized individuals as suffering from depression, 53–62 categorized as mild depression, 63–72 categorized as moderate depression, and 73 or more categorized as severe depression (19). Higher scores indicated more severe anxiety or depression.

The Eysenck Personality Questionnaire (EPQ) was used to assess the personality traits. The EPQ includes four sub-scales: internal and external tilt scale (EPQ -E), emotional scale (EPQ-N), psychological metamorphosis scale (EPQ-P, also known as mental quality) and efficacy scale (EPQ-L).

The Social Support Rate Scale (SSRS) was used to measure the type and levels of social support. It comprises of 10 items, three items that assess objective support (SSRS-ob), four items that assess subjective support (SSRS-sub), and three items that assess social support availability (SSRS-use).

The Life event scale (LES) contains 48 common life events, that are divided into 3 types such as family life events, events correlated with work and study, or social and other aspects. The events are mainly divided into positive events (LES-pos) and negative events (LES-neg). The higher the total score of LES, the greater mental stress the individual bears.

All scales used in this study are Chinese versions, which have been widely used in Chinese populations showing high reliability and validity.

### Statistical Analysis

The statistical analysis was conducted using SPSS 22.0 software. All *p*-values were two-tailed, reaching a significant level of 0.05. Continuous variables were represented by means and standard deviations, and categorical variables were represented by numbers and percentages. We used non-parametric test for continuous variable, and chi-square test for categorical variable and rank variable to compare the demographic and clinical variables between participants with or without anxiety or depression. Then, multivariate ordered logistic regression analysis were used to further explore the relationships between associated factors with anxiety or depression.

## Results

### Demographic and Clinical Characteristics

A total of 2,105 patients with physical diseases participated in this study, with an average age of 48.11 ± 13.43 years. One thousand thirty-eight (49.3%) of the participants were males and 1,067 (50.7%) were females. The majority of patients were married (*n* = 1,666, 79.1%), bachelor degree or junior college (*n* = 743, 35.3%), and had monthly incomes ranging from 3,000 to 5,000 (*n* = 846, 40.2%). Detailed demographic characteristics were listed (Table 1).

**Table 1:** Demographic and clinical characteristic of all patients (*n* = 2,105).

	**Frequency, *n* (%)**
Age (years), mean (SD)	48.11 ± 13.43
Gender	
Male	1,038 (49.3%)
Female	1,067 (50.7%)
Marital status	
Married	1,666 (79.1%)
Divorced/widowed	220 (10.5%)
Single	219 (10.4%)
Level of education	
Junior high school or below	570 (27.1%)
Senior high school or technical secondary school	682 (32.4%)
Bachelor degree or junior college	743 (35.3%)
Master degree or above	110 (5.2%)
Monthly income	
Under 1,000	111 (5.3%)
1,000–3,000	533 (25.3%)
3,000–5,000	846 (40.2%)
5,000–10,000	492 (23.4%)
Above 10,000	123 (5.8%)
Source of participants	
Outpatient	1,451 (68.9%)
Inpatient	654 (31.1%)
Diagnose of physical disease	
Multiple diseases	212 (10.1%)
Circulatory diseases	569 (27%)
Neurological diseases	375 (17.8%)
Digestive diseases	299 (14.2%)
Endocrine diseases	196 (9.3%)
Urogenital diseases	139 (6.6%)
Musculoskeletal diseases	65 (3.1%)
Respiratory diseases	63 (3%)
Cancer	50 (2.4%)
Other diseases	137 (6.5%)
Severity of physical disease	
Mild	1,037 (49.3%)
Moderate	914 (43.4%)
Severe	154 (7.3%)
Duration of physical diseases (years), mean (SD)	5.09 ± 5.35
EPQ score, mean (SD)
EPQ-E	53.59 ± 11.56
EPQ-N	50.73 ± 11.87
EPQ-P	72.69 ± 9.91
SSRS score, mean (SD)	
SSRS-ob	8.88 ± 2.81
SSRS-sub	19.62 ± 4.53
SSRS-use	6.59 ± 1.78
LES score, mean (SD)	
LES-neg	19.88 ± 27.10
LES-pos	5.85 ± 12.55
SAS score, mean (SD)	53.52 ± 10.47
SDS score, mean (SD)	60.09 ± 11.34

*The demographic and clinical characteristic of all patients. EPQ, the Eysenck Personality Questionnaire, EPQ-E, EPQ-N, EPQ-P represent EPQ three sub-scales; SSRS, the Social Support Rating Scale, SSRS-ob, SSRS-sub, SSRS-use represent three measurable dimensions of social support in SSRS (subjective support, objective support and utilization of support); LES, the Life Events Scale, LES-neg, and LES-pos represent negative life events and positive life events; SAS, the self-rating anxiety scale; SDS, the self-rating depression scale*.

68.9% (*n* = 1,451) participants were outpatients. Most participants (*n* = 569, 27.0%) had received a diagnosis of circulatory diseases, followed by neurological (*n* = 375, 17.8%), digestive (*n* = 299, 14.2%), endocrine (*n* = 196, 9.3%), urogenital (*n* = 139, 6.6%), musculoskeletal (*n* = 65, 3.1%), respiratory diseases (*n* = 63, 3%), and cancer (*n* = 50, 2.4%). The number of participants suffered from other diseases (infectious, hematological and immune diseases etc.) was very small, so we counted them together (*n* = 137, 6.5%). In addition, 212 (10.1%) participants had two or more co-morbidities (mainly hypertension, diabetes and COPD). The average duration of physical diseases was 5.09 ± 5.35 years. About the physical disease severity of the participants, most (*n* = 1,037, 49.3%) of the participants considered the severity of their physical diseases were mild, 914 (43.4%) were moderate, and 154 (7.3%) were severe (Table 1).

The mean scores for the EPQ three sub-scales, SSRS three sub-scales and two LES scores were 53.59 ± 11.56, 50.73 ± 11.87, and 72.69 ± 9.91; 8.88 ± 2.81, 19.62 ± 4.53, and 6.59 ± 1.78; 19.88 ± 27.10 and 5.85 ± 12.55, respectively. And the mean scores for the SAS and SDS were 53.52 ± 10.47 and 60.09 ± 11.34 (Table 1).

Fifty-one percentage circulatory, 52.5% neurological, 65.6% digestive, 50.5% endocrine, and 52.5% urogenital diseases patients considered themselves having mild severity of physical disease. And 54.7% multiple diseases, 60% musculoskeletal, 52.4% respiratory, and 51.8% other diseases patients thought themselves being moderate severity. Meanwhile, 50% cancer patients chose severe severity (Supplementary Table 1).

### Proportion of Anxiety and Depression

The proportion of anxiety among the patients with physical diseases was 63.3% (*n* = 1,332), most of them were mild (*n* = 630, 47.3%) and moderate (*n* = 566, 42.5%), and 136 (10.2%) were severe. The proportion of depression was 75.1% (*n* = 1,580), including 675 (42.7%) mild, 646 (40.9%) moderate, and 259 (16.4%) severe. Thousand two hundred and two (57.1%) patients had both anxiety and depression, accounted for 90.2% (1,202/1,332) anxious patients and 76.1% (1,202/1,580) depressive patients. Three hundred and ninety-five (18.8%) of the participants had no anxiety/depression, that is 81.2% patients had either anxiety or depression (Table 2).

**Table 2:** Proportion and severity of anxiety and depression in all patients (*n* = 2,105).

	**No-depression**	**Depression**				**ALL**
		**Mild**	**Moderate**	**Severe**	**All depression**	
No-anxiety	395	259	111	8	378	773
Anxiety						
Mild	72	264	262	32	558	630
Moderate	56	126	232	152	510	566
Severe	2	26	41	67	134	136
All anxiety	130	416	535	251	1,202	1,332
All	525	675	646	259	1,580	2,105

*The count of patients with anxiety and/or depression. The detailed proportion was not listed*.

We found that the proportion of anxiety among patients with different physical diseases varied from 41% (multiple diseases) to 80% (cancer), depression varied from 61.5% (musculoskeletal diseases) to 84% (cancer), and comorbidity anxiety with depression varied from 34% (multiple diseases) to 76% (cancer). The proportion of anxiety among inpatients was 30.9% (multiple diseases) to 90% (digestive diseases), outpatients was 42.5% (respiratory diseases) to 88% (cancer). And the proportion of depression was 46.7% (musculoskeletal diseases) to 85% (digestive diseases), outpatients was 69.7% (other diseases) to 88% (cancer). The proportion of comorbidity anxiety with depression was 22.8% (multiple diseases) to 85% (digestive diseases) among inpatients, and 42.5% (respiratory diseases) to 84% (cancer) among outpatients (Supplementary Table 2).

To sum up, the majority of patients with cancer showed severe anxious and depressive. Conversely, patients with multiple diseases mostly showed no anxious or depression. The severity of anxiety or depression in patients with various physical diseases were listed (Table 3).

**Table 3:** Severity of anxiety and depression in patients with various diseases.

	**Anxiety (** ***n*** **=** **2,105)**				**Depression (** ***n*** **=** **2,105)**			
	**No-anxiety,** ***n* (%)**	**Mild,** ***n* (%)**	**Moderate,** ***n* (%)**	**Severe,** ***n* (%)**	**No-depression,** ***n* (%)**	**Mild,** ***n* (%)**	**Moderate,** ***n* (%)**	**Severe,** ***n* (%)**
Multiple diseases	125 (59)	47 (22.2)	30 (14.2)	10 (4.7)	81 (38.2)	73 (34.4)	41 (19.3)	17 (8)
Circulatory diseases	138 (24.3)	182 (32)	204 (35.9)	45 (7.9)	122 (21.4)	163 (28.6)	195 (34.3)	89 (15.6)
Neurological diseases	175 (46.7)	109 (29.1)	67 (17.9)	24 (6.4)	106 (28.3)	135 (36)	98 (26.1)	36 (9.6)
Digestive diseases	96 (32.1)	111 (37.1)	74 (24.7)	18 (6)	63 (21.1)	101 (33.8)	107 (35.8)	28 (9.4)
Endocrine diseases	66 (33.7)	55 (28.1)	66 (33.7)	9 (4.6)	38 (19.4)	59 (30.1)	69 (35.2)	30 (15.3)
Urogenital diseases	45 (32.4)	41 (29.5)	44 (31.7)	9 (6.5)	25 (18)	48 (34.5)	54 (38.8)	12 (8.6)
Musculoskeletal diseases	28 (43.1)	19 (29.2)	15 (23.1)	3 (4.6)	25 (38.5)	18 (27.7)	15 (23.1)	7 (10.8)
Respiratory diseases	32 (50.8)	17 (27)	9 (14.3)	5 (7.9)	17 (27)	26 (41.3)	12 (19)	8 (12.7)
Cancer	10 (20)	18 (36)	13 (26)	9 (18)	8 (16)	14 (28)	14 (28)	14 (28)
Other diseases	58 (42.3)	31 (22.6)	44 (32.1)	4 (2.9)	40 (29.2)	38 (27.7)	41 (29.9)	18 (13.1)

*The count and proportion of anxiety and depression in patients with various physical diseases. Multiple diseases: patients combined with several chronic diseases, such as hypertension, coronary heart disease and diabetes etc. Other diseases: including infectious, hematological and immune diseases etc*.

### Associated Factors Analysis

We compared the demographic and clinical characteristics between anxiety and no-anxiety group. Compared with non-anxiety group, anxiety group had more females, divorced or widowed, highly educated participants, and had lower EPQ-N, EPQ-P, SSRS three sub-scale scores with higher LES two sub-scale scores (all *p*-value < 0.05). Besides, the diagnose and severity of physical disease had a significant difference between anxiety and no-anxiety groups (all *p*-value < 0.05). There was no significant difference in age, monthly income, EPQ-E score, and duration of physical disease (Table 4).

**Table 4:** Related factors of anxiety and depression (*n* = 2,105).

	**No-anxiety**	**Anxiety**	***p-*value**	**No-depression**	**Depression**	***P-*value**
Age (years), mean ± SD	47.33 ± 14.34	48.56 ± 12.86	*p* = 0.099	47.76 ± 14.1	48.22 ± 13.21	*p* = 0.919
Gender, *n* (%)						
Male	415 (53.7%)	623 (46.8%)	***p*** **=** 0.002^**^	304 (57.9%)	734 (46.5%)	***p*** **<** 0.001^***^
Female	358 (46.3%)	709 (53.2%)		221 (42.1%)	846 (53.5%)	
Marital status, *n* (%)						
Married	626 (81%)	1,040 (78.1%)	***p*** **<** 0.001^***^	419 (79.8%)	1,247 (78.9%)	***p*** **<** 0.001^***^
Divorced/widowed	49 (6.3%)	171 (12.8%)		33 (6.3%)	187 (11.8%)	
Single	98 (12.7%)	121 (9.1%)		73 (13.9%)	146 (9.2%)	
Level of education, *n* (%)						
Junior high school or below	237 (30.7%)	333 (25%)	***p*** **=** 0.022^*^	128 (24.4%)	442(28%)	*p* = 0.073
Senior high school or technical secondary school	237 (30.7%)	445 (33.4%)		167 (31.8%)	515(32.6%)	
Bachelor degree or junior college	263 (34%)	480 (36%)		204 (38.9%)	539(34.1%)	
Master degree or above	36 (4.7%)	74 (5.6%)		26 (5%)	84(5.3%)	
Monthly income, *n* (%)						
Under 1,000	45 (5.8%)	66 (5%)	*p* = 0.063	20 (3.8%)	91 (5.8%)	***p*** **<** 0.001^***^
1,000–3,000	165 (21.3%)	368 (27.6%)		79 (15%)	454 (28.7%)	
3,000–5,000	324 (41.9%)	522 (39.2%)		229 (43.6%)	617 (39.1%)	
5,000–10,000	200 (25.9%)	292 (21.9%)		163 (31%)	329 (20.8%)	
Above 10,000	39 (5%)	84 (6.3%)		34 (6.5%)	89 (5.6%)	
Diagnose of physical disease, *n* (%)						
Multiple diseases	125 (16.2%)	87 (6.5%)	***p*** **<** 0.001^***^	81 (15.4%)	131 (8.3%)	***p*** **<** 0.001^***^
Circulatory diseases	138 (17.9%)	431 (32.4%)		122 (23.2%)	447 (28.3%)	
Neurological diseases	175 (22.6%)	200 (15%)		106 (20.2%)	269 (17%)	
Digestive diseases	96 (12.4%)	203 (15.2%)		63 (12%)	236 (14.9%)	
Endocrine diseases	66 (8.5%)	130 (9.8%)		38 (7.2%)	158 (10%)	
Urogenital diseases	45 (5.8%)	94 (7.1%)		25 (4.8%)	114 (7.2%)	
Musculoskeletal diseases	28 (3.6%)	37 (2.8%)		25 (4.8%)	40 (2.5%)	
Respiratory diseases	32 (4.1%)	31 (2.3%)		17 (3.2%)	46 (2.9%)	
Cancer	10 (1.3%)	40 (3%)		8 (1.5%)	42 (2.7%)	
Other diseases	58 (7.5%)	79 (5.9%)		40 (7.6%)	97 (6.1%)	
Duration of physical diseases (years), mean ± SD	5.09 ± 5.27	5.09 ± 5.4	*p* = 0.747	5.17 ± 5.44	5.06 ± 5.32	*p* = 0.825
Severity of physical disease, *n* (%)						
Mild	425 (55%)	612 (45.9%)	***p*** **<** 0.001^***^	265 (50.5%)	772 (48.9%)	*p* = 0.551
Moderate	297 (38.4%)	617 (46.3%)		222 (42.3%)	692 (43.8%)	
Severe	51 (6.6%)	103 (7.7%)		38 (7.2%)	116 (7.3%)	
EPQ score, mean ± SD						
EPQ-E	53.97 ± 12.06	53.36 ± 11.26	*p* = 0.189	49.91 ± 11.54	54.81 ± 11.31	***p*** **<** 0.001^***^
EPQ-N	55.8 ± 11.7	47.78 ± 10.93	***p*** **<** 0.001^***^	55.27 ± 11.63	49.21 ± 11.56	***p*** **<** 0.001^***^
EPQ-P	75.69 ± 9.18	70.95 ± 9.9	***p*** **<** 0.001^***^	75.31 ± 9.08	71.82 ± 10.02	***p*** **<** 0.001^***^
SSRS score, mean ± SD						
SSRS-ob	9.31 ± 2.75	8.64 ± 2.81	***p*** **<** 0.001^***^	9.82 ± 2.82	8.57 ± 2.73	***p*** **<** 0.001^***^
SSRS-sub	20.82 ± 4.71	18.93 ± 4.27	***p*** **<** 0.001^***^	21.52 ± 4.54	18.99 ± 4.35	***p*** **<** 0.001^***^
SSRS-use	6.97 ± 1.76	6.37 ± 1.75	***p*** **<** 0.001^***^	7.45 ± 1.88	6.3 ± 1.65	***p*** **<** 0.001^***^
LES score, mean ± SD						
LES-neg	12.87 ± 22.56	23.94 ± 28.64	***p*** **<** 0.001^***^	14.4 ± 27.56	21.7 ± 26.7	***p*** **<** 0.001^***^
LES-pos	4.37 ± 10.18	6.71 ± 13.68	***p*** **<** 0.001^***^	6.05 ± 13.63	5.78 ± 12.18	*p* = 0.918

*The associated factors of anxiety and depression by chi-square test and non-parametric test. The significance level*:

* *p < 0.05*,

** *p < 0.01*,

*** *p < 0.001*.

*The bold values mean p < 0.05*.

Statistical analysis showed that the depression group had more females, divorced or widowed, and low-income participants, with higher EPQ-E and LES-neg scores, and lower EPQ-N, EPQ-P, three SSRS sub-scales scores. There was also a significant difference in patients with different physical diseases between depression and no-depression groups (all *p*-value < 0.05). The age, level of education, duration and severity of physical diseases, as well as LES-pos had no significant difference between depression and no-depression groups (Table 4).

Finally, we analyzed association of demographic, clinical, social, psychological factors with anxiety symptoms (no anxiety symptoms, mild anxiety symptoms, moderate anxiety symptoms and severe anxiety symptoms) and depressive symptoms (no depressive symptoms, mild depressive symptoms, moderate depressive symptoms, and severe depressive symptoms). Our results showed that elders (OR_anxiety_ 1.02, 95% CI 1.01–1.03) were more likely to exhibit more anxious, while married (OR_depression_ 2.00, 95% CI 1.42–2.81) and divorced/widowed (OR_depression_ 1.98, 95% CI 1.29–3.05) patients were more depressive than singles. In addition, males (OR_anxiety_ 0.58, 95% CI 0.48–0.69 vs. OR_depression_ 0.63, 95% CI 0.53–0.76) exhibited slighter symptoms in both anxiety and depression than females. Patients with degree of junior high school or below (OR_anxiety_ 0.54, 95% CI 0.34–0.86), monthly income from 3,000 to 5,000 (OR_anxiety_ 0.65, 95% CI 0.44–0.94) showed slighter anxiety than patients with higher education and income. However, patients with monthly income from 1,000 to 3,000 (OR_depression_ 1.56, 95% CI 1.05–2.31) showed more severe depressive than patients with higher income (Table 5).

**Table 5:** Factors associated with anxiety and depression by multivariate ordered logistic regression.

	**Anxiety**			**Depression**		
	**OR**	**95% CI**	***P-*value**	**OR**	**95% CI**	***P-*value**
Age (years), mean (SD)	**1.02**	**(1.01–1.03)**	<0.001^***^	1.00	(0.99–1.01)	0.482
Gender						
Male	**0.58**	**(0.48–0.69)**	<0.001^***^	**0.63**	**(0.53**–**0.76)**	<0.001^***^
Female	(reference)			(reference)		
Marital status						
Married	1.24	(0.87–1.75)	0.233	**2.00**	**(1.42**–**2.81)**	<0.001^***^
Divorced/widowed	1.44	(0.93–2.22)	0.104	**1.98**	**(1.29**–**3.05)**	0.002^**^
Single	(reference)			(reference)		
Level of education						
Junior high school or below	**0.54**	**(0.34**–**0.86)**	0.009^**^	0.72	(0.46–1.12)	0.147
Senior high school or technical secondary school	0.73	(0.48–1.12)	0.151	0.90	(0.59–1.36)	0.605
Bachelor degree or junior college	0.82	(0.55–1.22)	0.329	0.77	(0.52–1.14)	0.190
Master degree or above	(reference)			(reference)		
Monthly income						
Under 1,000	0.73	(0.43–1.23)	0.238	1.41	(0.84–2.37)	0.189
1,000–3,000	0.79	(0.53–1.18)	0.255	**1.56**	**(1.05**–**2.31)**	0.028^*^
3,000–5,000	**0.65**	**(0.44**–**0.94)**	0.023^*^	0.95	(0.66–1.38)	0.785
5,000–10,000	0.72	(0.49–1.06)	0.098	0.91	(0.62–1.33)	0.623
Above 10,000	(reference)		.	(reference)		
Diagnose of physical disease						
Multiple diseases	**0.54**	**(0.34**–**0.85)**	0.007^**^	**0.61**	**(0.4**–**0.94)**	0.024^*^
Circulatory diseases	**1.99**	**(1.36**–**2.91)**	<0.001^***^	1.38	(0.95–2.01)	0.087
Neurological diseases	1.41	(0.94–2.11)	0.096	**1.49**	**(1.01**–**2.21)**	0.047^*^
Digestive diseases	1.35	(0.91–2.01)	0.140	1.35	(0.92–2)	0.130
Endocrine diseases	1.33	(0.86–2.06)	0.196	**1.56**	**(1.02**–**2.4)**	0.040^*^
Urogenital diseases	1.48	(0.93–2.34)	0.098	1.16	(0.74–1.83)	0.516
Musculoskeletal diseases	0.94	(0.53–1.67)	0.832	0.76	(0.43–1.34)	0.340
Respiratory diseases	**0.51**	**(0.27**–**0.94)**	0.031^*^	0.57	(0.31–1.02)	0.060
Cancer	**2.00**	**(1.05**–**3.83)**	0.036^*^	**2.01**	**(1.05**–**3.84)**	0.035^*^
Other diseases	(reference)			(reference)		
Severity of physical disease						
Mild	0.77	(0.54–1.1)	0.155	1.06	(0.74–1.5)	0.762
Moderate	1.15	(0.82–1.63)	0.426	1.26	(0.9–1.77)	0.181
Severe	(reference)			(reference)		
Duration of physical diseases (years), mean (SD)	1.00	(0.98–1.01)	0.652	1.01	(0.99–1.03)	0.190
EPQ score, mean (SD)						
EPQ-E	**0.98**	**(0.97–0.99)**	<0.001^***^	**1.01**	**(1.00–1.02)**	0.014^*^
EPQ-N	**0.96**	**(0.95–0.96)**	<0.001^***^	**0.96**	**(0.95–0.97)**	<0.001^***^
EPQ-P	**0.95**	**(0.94–0.96)**	<0.001^***^	**0.97**	**(0.96–0.97)**	<0.001^***^
SSRS score, mean (SD)						
SSRS-ob	0.98	(0.94**–**1.01)	0.189	**0.91**	**(0.88–0.94)**	<0.001^***^
SSRS-sub	**0.95**	**(0.93–0.98)**	<0.001^***^	**0.97**	**(0.95–0.99)**	0.003^**^
SSRS-use	**0.91**	**(0.86–0.97)**	0.001^**^	**0.82**	**(0.77–0.86)**	<0.001^***^
LES score, mean (SD)						
LES-neg	**1.01**	**(1.01–1.01)**	<0.001^***^	**1.02**	**(1.01–1.02)**	<0.001^***^
LES-pos	1.00	(1**–**1.01)	0.256	**0.98**	**(0.98–0.99)**	<0.001^***^

*All factors of anxiety and depression by multivariate ordered logistic regression. The significance level*:

* *p < 0.05*,

** *p < 0.01*,

*** *p < 0.001*.

*The bold values mean p < 0.05*.

We explored that whether the physical diseases effected the anxiety and depressive symptoms. The results showed that patients with multiple diseases (OR_anxiety_ 0.54, 95% CI 0.34–0.85 vs. OR_depression_ 0.61, 95% CI 0.4–0.94) exhibited slighter symptoms in both anxiety and depression than other diseases, while patients with respiratory diseases (OR_anxiety_ 0.51, 95% CI 0.27–0.94) only showed slighter anxiety. In addition, we found that patients with circulatory diseases (OR_anxiety_ 1.99, 95% CI 1.36–2.91) showed more severe anxiety. Besides, patients with cancer (OR_anxiety_ 2.00, 95% CI 1.05–3.83 vs. OR_depression_ 2.01, 95% CI 1.05–3.84) showed more severe symptoms in both anxiety and depression than patients with other diseases, while patients with neurological diseases (OR_depression_ 1.49, 95% CI 1.01–2.21) and endocrine diseases (OR_depression_ 1.56, 95% CI 1.02–2.4) only showed more severe depression (Table 5).

We found that high EPQ-N (OR_anxiety_ 0.96, 95% CI 0.95–0.96 vs. OR_depression_ 0.96, 95% CI 0.95–0.97), EPQ-P (OR_anxiety_ 0.95, 95% CI 0.94–0.96 vs. OR_depression_ 0.97, 95% CI 0.96–0.97), SSRS-sub (OR_anxiety_ 0.95, 95% CI 0.93–0.98 vs. OR_depression_ 0.97, 95% CI 0.95–0.99), SSRS-use (OR_anxiety_ 0.91, 95% CI 0.86–0.97 vs. OR_depression_ 0.82, 95% CI 0.77–0.86) were inversely associated with both anxiety and depression. Negative life events (OR_anxiety_ 1.01, 95% CI 1.01–1.01 vs. OR_depression_ 1.02, 95% CI 1.01–1.02) were significantly associated with both anxiety and depression. Furthermore, high SSRS-ob and LES-pos scores were inversely associated with depression. However, patients with high EPQ-E score showed different tendency in anxiety and depression (OR_anxiety_ 0.98, 95% CI 0.97–0.99 vs. OR_depression_ 1.01, 95% CI 1.00–1.02) (Table 5).

## Discussion

It has become a consensus that anxiety and depression have a low recognition rate in general hospitals. Besides, patients with physical diseases have a high prevalence of anxiety and depression in various clinical departments. But the results obtained in previous studies are quite different. The present study is the first large-scale field survey in general hospitals, which could reflect the clinical problems better. The survey used a consecutive sample, including outpatients and inpatients from all the clinical departments, the sampling strategies could avoid the selective bias. Otherwise, We chose the patients tending to exhibit anxiety or depression, focusing on the high-risk group for anxiety and depression.

Totally, 81.2% patients had either anxiety or depression, which meant the medical staff in general hospitals could use the HADS to screening anxiety and depression in patients with physical problems. A time-point survey conducted that, compared with anxiety, the incidence of depression in hospitalized patients (7). Our survey further confirmed the point, the results showed that 45.7% inpatients had anxiety, and 61.6% inpatients had depression. However, no study has investigated anxiety and depression in outpatients in Chinese general hospitals before, while the outpatients accounted for the majority of patients in general hospitals. The outpatients were lack of professional observation by doctors, which meant their symptoms of anxiety or depression could influence the physical diseases more seriously. Our study indicated that the proportion of depression in outpatients was also higher than anxiety. Furthermore, it is suggested that depression is more common than anxiety in patients with physical diseases in all the various clinical departments, which was similar to another research (20). In addition, above 90% anxious patients exhibited depression. Medical staff could focus more on the depression. We also found that outpatients had a higher proportion of both anxiety and depression than inpatients. Receiving systemic treatment and understanding of the condition might be part of the reason. The result suggested that the outpatients needed more concern to treat their anxious and depression. Additionally, we found that comorbidity anxiety and depression accounted for the majority of anxiety or depression patients. A recent systematic review found robust and consistent evidence of comorbidity between broadly defined mood and anxiety disorders, and there are several speculations about the reasons for the high comorbidity of anxiety and depression (21). The reason of comorbidity between anxiety and depressive disorders has multiple interpretations. For example, Middeldorp et al. found that comorbidity between anxiety and depressive disorder may be explained by a shared genetic vulnerability, which was supported by some family studies, while others suggest that comorbidity may be due to one disorder being an epiphenomenon of the other (22).

The associated factors for anxiety and depression were studied by means of single factor analysis and multivariate analysis. Our findings showed that females, patients with cancer, negative life events and poor social supports were more likely to feel anxious or depressed. Advanced age, high education and income were associated with anxiety symptoms, while patients, who were married, divorced or widowed, and with low income, were more likely to exhibit depression. In addition, positive life events might decrease the risk of depression. Our main findings were consistent with previous studies (9–11, 13). Furthermore, our result showed that high EPQ-N and EPQ-P scores were inversely associated with anxiety and depression, which meant patients who exhibited lower personality traits of sociability and expressive instability were less likely to exhibit anxiety and depression. According to the results, we suspect that social pressure might be the source of anxiety and depression in patients with physical diseases. Interestingly, patients with high EPQ-E score showed different tendency in anxiety and depression, which meant that external character showed slighter anxiety and more severe depression. The result indicated that outgoing ones could reduce their anxious symptom by social activity or other ways, while the depression could not be released, even worse. Therefore, for patients with external character, we prefer to focus on their condition of depression symptom.

Moreover, we found that cancer patients had a higher proportion of both anxiety and depression compared to other physical diseases. After using multivariate ordered logistic regression analysis, the risk of anxiety and depression in cancer patients was still higher than other diseases, suggesting that anxiety and depression are more common in cancer participants than other diseases. Furthermore, specific physical diseases (except respiratory diseases) were at a higher risk of anxiety and depression than multiple diseases. The reason might be that the multiple diseases patients mainly suffered from chronic diseases, such as hypertension, COPD, coronary artery disease etc. Otherwise, we found that patients with circulatory diseases showed more severe anxiety, and patients with neurological diseases and endocrine diseases showed more severe depression. Other researches also found that physical diseases patients were at a high risk of anxiety and depression. For example, Yan et al. conducted a study in Shanghai, China. They found that the prevalence of anxiety and depression in cancer survivors was 28.2 and 48.2%, respectively (23). Another study found that the prevalence of anxiety and depression in cancer patients was 52 and 48%, respectively (24). According to the results of previous studies, the prevalence of depression varies from ~4 to 49% (25). A cross-sectional hospital-based study among cardiac patients in a Palestinian population showed that the overall proportion was 52.9 and 53.1% for the presence of depressive and anxiety symptoms (26). The study of López-Espuela et al. found that depression was present in 42.2% patients at 6 months after stroke (27). Zhou et al. found that 21.2% of the ischemic stroke survivors were depressed and 23.5% were anxious (28). Compared with other surveys, our results showed a higher proportion than other researches. This was, perhaps, because we selected patients with possible depression or anxiety. Besides, the scales (SAS, SDS) used here included somatic symptoms, meanwhile all of our participants were with physical diseases, which might raise the scores of the scales.

There are several limitations that should be mentioned in our study. First, both SAS and SDS were self-evaluated scales, and their scoring criteria include physical symptoms, so the reliability of SAS and SDS in patients with physical diseases needs further study. Second, although physical diseases have been shown to increase the risk of anxiety and depression, there is evidence that anxiety disorders have a higher burden of physical comorbidity (29). For example, Hesdorffer et al. found that there was a bidirectional relationship between psychiatric disorders and neurological diseases, which means that mood disorders might make physical diseases worse and vice versa (30). And this might make patients exhibit more severe physical diseases. Third, our cross-sectional study cannot determine the time of the mood disorders and physical diseases occurrence. Physical diseases might be the result of anxiety or depression.

## Conclusions

Our results indicate that patients with physical diseases have a high rate of anxiety and depression, especially cancer patients. Besides, patients with anxiety are at high risk for depression, and vice versa. Gender, marital status, personality traits, social supports and life events are also the risk factors for anxiety and depression. Timely intervention of emotional disorders in patients with physical diseases is helpful to their treatment and recovery.

## Limitations

There are several limitations that should be mentioned in our study. First, both SAS and SDS were self-evaluated scales, and include items related with physical symptoms, so the reliability of SAS and SDS in patients with physical diseases needs further study. Second, the population of the patients in the same period was unknown, the incidence of anxiety and depression could not be calculated. However, there were other surveys reported the data of incidence. Third, the cross-sectional study cannot determine the timing of the anxiety or depression and physical diseases occurring, physical diseases might be the result of anxiety or depression. We did not distinguish the physical diseases and the physical symptom caused by anxiety or depression.

## Data Availability Statement

The datasets presented in this article are not readily available because the data are not publicly available due to privacy or ethical restrictions. Requests to access the datasets should be directed to Gaohua Wang, wgh6402@163.com.

## Ethics Statement

The studies involving human participants were reviewed and approved by Ethics Committee of Renmin Hospital of Wuhan University (No. 2012-031). Written informed consent to participate in this study was provided by the participants' legal guardian/next of kin.

## Author Contributions

GW, HW, LX, and ZL designed the study and wrote the protocol. HW, LX, HH, ZY, CN, YX, and WY performed the experiments. WY analyzed the data and wrote the manuscript. HH, HW, and GW revised the manuscript. All authors contributed to and have approved the final manuscript.

## Conflict of Interest

The authors declare that the research was conducted in the absence of any commercial or financial relationships that could be construed as a potential conflict of interest.

## Publisher's Note

All claims expressed in this article are solely those of the authors and do not necessarily represent those of their affiliated organizations, or those of the publisher, the editors and the reviewers. Any product that may be evaluated in this article, or claim that may be made by its manufacturer, is not guaranteed or endorsed by the publisher.
