# Supplementary material for: Anxiety and Depression in Patients With Physical Diseases and Associated Factors: A Large-Scale Field Survey in General Hospitals in China
Source: Front Psychiatry. 2021 Jul 28;12:689787. doi: 10.3389/fpsyt.2021.689787 (PMC8359676; doi:10.3389/fpsyt.2021.689787)
Supplement: Supplementary file 1 [file Data_Sheet_1.docx]

| **Supplying Table 1. Severity of physical diseases among patients with various diseases** | | | |
| --- | --- | --- | --- |
|  | **severity** | | |
|  | **mild** | **moderate** | **severe** |
| Multiple diseases | 77 (36.3%) | 116 (54.7%) | 19 (9%) |
| Circulatory diseases | 290 (51%) | 242 (42.5%) | 37 (6.5%) |
| Neurological diseases | 197 (52.5%) | 153 (40.8%) | 25 (6.7%) |
| Digestive diseases | 196 (65.6%) | 94 (31.4%) | 9 (3%) |
| Endocrine diseases | 99 (50.5%) | 93 (47.4%) | 4 (2%) |
| Urogenital diseases | 73 (52.5%) | 52 (37.4%) | 14 (10.1%) |
| Musculoskeletal diseases | 18 (27.7%) | 39 (60%) | 8 (12.3%) |
| Respiratory diseases | 28 (44.4%) | 33 (52.4%) | 2 (3.2%) |
| Cancer | 4 (8%) | 21 (42%) | 25 (50%) |
| Other diseases | 55 (40.1%) | 71 (51.8%) | 11 (8%) |

| **Supplying Table 2. Proportion of anxiety and depression among inpatients with various diseases (n=2105)** | | | | | | | | | |
| --- | --- | --- | --- | --- | --- | --- | --- | --- | --- |
|  | **All (n=2105)** | | | **Inpatient (n=654)** | | | **Outpatient (n=1451)** | | |
|  | **Anxiety,**  **N (%)** | **Depression,**  **N (%)** | **Co-morbidity anxiety with depression,**  **N (%)** | **Anxiety,**  **N (%)** | **Depression,**  **N (%)** | **Co-morbidity anxiety with depression,**  **N (%)** | **Anxiety,**  **N (%)** | **Depression,**  **N (%)** | **Co-morbidity anxiety with depression,**  **N (%)** |
| Multiple diseases | 87 (41%) | 131 (61.8%) | 72 (34%) | 50 (30.9%) | 90 (55.6%) | 37 (22.8%) | 37 (74%) | 41 (82%) | 35 (70%) |
| Circulatory diseases | 431 (75.7%) | 447 (78.6%) | 378 (66.4%) | 74 (63.2%) | 77 (65.8%) | 64 (54.7%) | 357 (79%) | 370 (81.9%) | 314 (69.5%) |
| Neurological diseases | 200 (53.3%) | 269 (71.7%) | 179 (47.7%) | 80 (38.5%) | 123 (59.1%) | 70 (33.7%) | 120 (71.9%) | 146 (87.4%) | 109 (65.3%) |
| Digestive diseases | 203 (67.9%) | 236 (78.9%) | 191 (63.9%) | 18 (90%) | 17 (85%) | 17 (85%) | 185 (66.3%) | 219 (78.5%) | 174 (62.4%) |
| Endocrine diseases | 130 (66.3%) | 158 (80.6%) | 116 (59.2%) | 13 (32.5%) | 27 (67.5%) | 13 (32.5%) | 117 (75%) | 131 (84%) | 103 (66%) |
| Urogenital diseases | 94 (67.6%) | 114 (82%) | 91 (65.5%) | 9 (81.8%) | 8 (72.7%) | 8 (72.7%) | 85 (66.4%) | 106 (82.8%) | 83 (64.8%) |
| Musculoskeletal diseases | 37 (56.9%) | 40 (61.5%) | 35 (53.8%) | 13 (43.3%) | 14 (46.7%) | 12 (40%) | 24 (68.6%) | 26 (74.3%) | 23 (65.7%) |
| Respiratory diseases | 31 (49.2%) | 46 (73%) | 27 (42.9%) | 14 (60.9%) | 13 (56.5%) | 10 (43.5%) | 17 (42.5%) | 33 (82.5%) | 17 (42.5%) |
| Cancer | 40 (80%) | 42 (84%) | 38 (76%) | 18 (72%) | 20 (80%) | 17 (68%) | 22 (88%) | 22 (88%) | 21 (84%) |
| Other diseases | 79 (57.7%) | 97 (70.8%) | 75 (54.7%) | 10 (55.6%) | 14 (77.8%) | 8 (44.4%) | 69 (58%) | 83 (69.7%) | 67 (56.3%) |
